# Probiotic as Adjuvant Significantly Improves Protection of the Lanzhou Trivalent Rotavirus Vaccine against Heterologous Challenge in a Gnotobiotic Pig Model of Human Rotavirus Infection and Disease

**DOI:** 10.3390/vaccines10091529

**Published:** 2022-09-14

**Authors:** Viviana Parreno, Muqun Bai, Fangning Liu, Jiqiang Jing, Erika Olney, Guohua Li, Ke Wen, Xingdong Yang, Tammy Bui Castellucc, Jacob F. Kocher, Xu Zhou, Lijuan Yuan

**Affiliations:** 1 Department of Biomedical Sciences and Pathobiology, Virginia-Maryland College of Veterinary Medicine, Virginia Polytechnic Institute and State University, Blacksburg, VA 24061, USA; 2 INCUINTA, Institutot de Virología e Innovaciones Tecnológicas (IVIT), Instituto Nacional de Tecnología Agropecuaria (INTA)-CONICET, Ciudad Autónoma de Buenos Aires C1033AAE, Argentina; 3 Lanzhou Institute of Biological Products, Lanzhou 730046, China; 4 Department of Animal Medicine, Shandong Vocational College of Animal Husbandry and Veterinary Medicine, Weifang 261071, China; 5 College of Veterinary Medicine, Midwestern University, Glendale, AZ 85308, USA

**Keywords:** rotavirus vaccine, probiotic adjuvant, *Lactobacillus rhamnosus* GG, Lanzhou trivalent vaccine, gnotobiotic pig

## Abstract

This preclinical study in the gnotobiotic (Gn) pig model of human rotavirus (HRV) infection and disease evaluates the effect of probiotic *Lactobacillus rhamnosus* GG (LGG) as a mucosal adjuvant on the immunogenicity and cross-protective efficacy of the Lanzhou live oral trivalent (G2, G3, G4) vaccine (TLV, aka LLR3). Gn pigs were immunized with three doses of TLV with or without concurrent administration of nine doses of LGG around the time of the first dose of the TLV vaccination, and were challenged orally with the virulent heterotypic Wa G1P[8] HRV. Three doses of TLV were highly immunogenic and conferred partial protection against the heterotypic HRV infection. LGG significantly enhanced the intestinal and systemic immune responses and improved the effectiveness of protection against the heterotypic HRV challenge-induced diarrhea and virus shedding. In conclusion, we demonstrated the immune-stimulating effects of probiotic LGG as a vaccine adjuvant and generated detailed knowledge regarding the cross-reactive and type-specific antibody and effector B and T cell immune responses induced by the TLV. Due to the low cost, ease of distribution and administration, and favorable safety profiles, LGG as an adjuvant has the potential to play a critical role in improving rotavirus vaccine efficacy and making the vaccines more cost-effective.

## 1. Introduction

Rotavirus (RV) infection was responsible for more than 258 million episodes of diarrhea and around 128,500 deaths among children younger than 5 years throughout the world in 2016, according to a global burden reported in 2018 [1]. Of the total deaths, 104,733 were registered in sub-Saharan Africa. In China, RV is also a leading cause of severe diarrhea among children, causing over 40% of diarrhea hospitalizations and ~30% of diarrhea-related outpatient visits in children aged < 5 years [2]. Severe RV disease occurs early in life, with 14% of the hospitalizations occurring in children younger than 6 months of age, over half of the RV-related hospitalizations occurring in the first year of life, and 91% of RV related hospitalizations occurring by 2 years of age in China. These results highlight that the implementation of vaccination programs early in life has the highest potential to prevent the majority of the burden of severe RV disease [2].

Among the different RV vaccines that have been developed [3], the Lanzhou Lamb RV vaccine (LLR) is a live, attenuated oral vaccine consisting of serotype G10P[12] that has been licensed in China for domestic application since 2000. It was the only vaccine available in the private market before 2018, and was recommended for 2–36-month-old children, administered in one dose between 6 and 12 months of age followed by yearly boosters. More than 83 million doses of the LLR vaccine have been lot-released over the past 18 years (data collected from Vaccine Lot Release System of National Immunization Program, Chinese Center for Disease Control and Prevention, Beijing, China). Post-marketing effectiveness of the vaccine was evaluated in several hospital-based and population-based case-control studies, showing that LLR vaccine efficacy against RV gastroenteritis ranged from 35.0% against any-severity RVGE to 73.3% against severe diarrhea requiring hospitalization in China [4]. In addition, several metanalysis studies showed a 5% decrease in the RV gastroenteritis rate (from 45% in 2001–2005 to 40% in 2006–2011) and a 6% reduction in the RV detection rate in hospitalized patients from 2009–2011 (data from the China Viral Diarrhea Surveillance Network). However, due to the lack of reduction of RV detection rate in ambulatory patients and the emergence of different strains including the G9P[8] combination that became the prevalent strain in the recent year in China, an alternative improved RV vaccine was developed and manufactured by Lanzhou Institute of Biological Products Co., Ltd., (Lanzhou, China). The trivalent Lanzhou RV reassortant vaccine (TLV) consists of three rotavirus reassortants, LD, LS, and LH strains, that were generated using human RV (HRV) field isolate D36 (G2), S25 (G3) and the standard Hochi (G4) strain as VP7/VP4 gene donor strains and the Lanzhou lamb RV (LLR) strain (G10P[12]) as the backbone strain. The G2 and G4 reassortants each carry a single gene VP7 from the respective donor strain. The G3 reassortant carries two gene segments VP7 (G3) and VP4 (P[2]) from the donor strain. A multicentered double-blind, placebo-controlled phase III clinical trial, conducted from 2012 to 2014, indicated that the vaccine is highly immunogenic, safe, and gave a vaccine efficacy against RV gastroenteritis of any severity of 56.6%, 70.3% against severe RVGE, and 74.0% against hospitalization [5]. These efficacy results agreed with the results of phase III clinical trials of RotaRix (GlaxoSmithKline PLC, Middlesex, UK) and RotaTeq (Merck & Co., Inc. Rahway, NJ, USA) vaccines recently performed in China, giving 72% and 78.9% protection against severe RVGE, respectively [6,7].

It has been well recognized that microbiota and probiotics play a key role in shaping immune system maturation and activity in young children [8,9]. The use of probiotic bacteria as vaccine adjuvant represents a strategic key for further improvement of immunogenicity and efficacy of childhood vaccines, but more experimental evidence is still needed [10,11]. In our previous studies, we evaluated the adjuvant effect of several probiotic strains at different doses on an oral rotavirus vaccine in a gnotobiotic (Gn) pig model of HRV infection and diarrhea [12,13,14]. *Lactobacillus rhamnosus* GG (LGG) enhanced B- and T-cell immune responses induced by the Wa strain attenuated HRV (AttHRV) vaccine (similar strain as the G1P[8] Rotarix vaccine) and increased the protection rate against homologous challenge [14]. The study also found that different dosages of LGG preferentially promoted AttHRV vaccine-induced virus-specific B- and T-cell immune responses in terms of lymphoid tissue distribution (intestinal versus systemic), B-cell versus T-cell responses, and differences in protective efficacy against rotavirus diarrhea in Gn pigs. The administration of nine doses of LGG was the most effective in promoting innate immunity [12] and IFN-γ-producing effector CD4+ and CD8+ T-cell responses [14].

The Gn pig model of HRV infection, disease, and immunity is an excellent preclinical model to evaluate vaccine efficacy [15]. Gn pigs are free of exposure to extraneous viruses or bacteria, assuring that exposure to a single microbe/pathogen, and the specific immune responses induced, can be analyzed. The Gn status prevents both interference and confounding factors from wild swine rotavirus contaminations that are inevitable in conventional pig facilities. The microbiome-free state of the Gn pigs allows for their colonization with defined probiotic strains and permits analysis of the effect seen on the rotavirus vaccine as attributable to the probiotic (and not some extraneous ‘microflora’ bacteria). Gn pigs are immunocompetent at birth and are capable of generating robust immune responses upon viral/bacterial infection [16]. Similar to humans, pigs are monogastric and are physiologically, anatomically, and immunologically similar to humans [17]. The mucosal immune system of the pig closely resembles that of humans; thus, the pig provides a unique animal model for the study of innate and adaptive immunity related to human enteric viral diseases. Only neonatal pigs, and not conventional lab animals (mice, rats, guinea pigs, rabbits), are susceptible to diarrhea following HRV inoculation.

The present study aimed to evaluate the effect of the probiotic LGG as a mucosal adjuvant to improve the immunogenicity and cross-protective efficacy of the trivalent reassortant Lanzhou vaccine (TLV) against heterotypic challenge in the Gn pig model of HRV Wa strain (G1P[8]) infection and disease. The study presents an in-depth preclinical evaluation of the immunogenicity of the TLV in terms of the development of antibody-secreting cells (ASC) and IFN-γ CD4^+^ and CD8^+^ T-cell responses in intestinal and systemic tissues; moreover, the kinetics of serum IgG and IgA responses to G1, G2, G3, and G4 VP7 G-types are shown. The protective effects of the TLV, with or without the LGG adjuvant, against diarrhea and virus shedding upon heterotypic challenge with one of the most prevalent HRV strains (G1P[8]) circulating worldwide were demonstrated.

## 2. Materials and Methods

### 2.1. Vaccine

**Figure 1:**
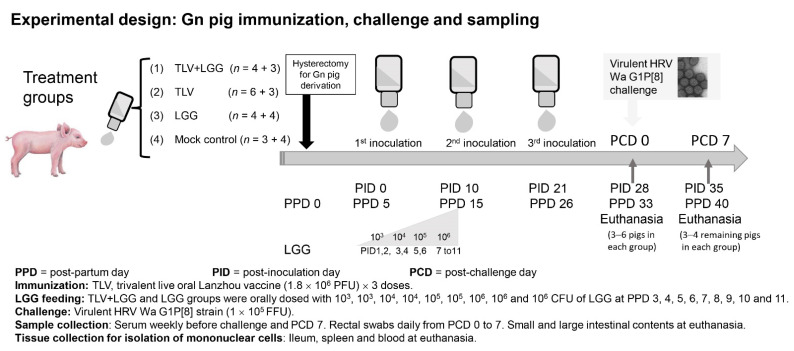
Experimental design: Gnotobiotic (Gn) pig immunization, *Lactobacillus rhamnosus* (LGG) feeding, challenge, and sampling.

TLV, the trivalent rotavirus reassortant (human x lamb) vaccine, was manufactured by the Lanzhou Institute of Biological Production, China. The TLV is composed of three rotavirus reassortant strains LD9 (G2P[12]), LS4/9(G3P[2]), and LH9 (G4P[12]) that were generated using human rotavirus field isolates D36 (G2), S25 (G3) and the standard Hochi (G4) [18] strains as VP7 gene donor strains and the Lanzhou lamb rotavirus (LLR) strain (G10P[12]) as the parental strain. The G2 and G4 reassortants each carried a single gene VP7 from the respective donor strain. The G3 reassortant carried two gene segments, VP7 (G3) and VP4 (P[2]), from the donor strain. For the immunization of the Gn pigs, the animals received three oral doses of vaccine, containing a total virus titer of 1.8 × 10^6^ plaque forming units (PFU) Animals were immunized at 5 days of age (post-inoculation day 0 (PID 0), 15 days of age (PID 10), and 26 days of age (PID 21) (Figure 1).

### 2.2. Probiotic Bacteria Used as Vaccine Adjuvant

*Lactobacillus rhamnosus* GG (ATCC 53103) were propagated in Lactobacilli MRS broth (Weber Scientific, Hamilton Township, NJ, USA) overnight at 37 °C anaerobically (85% nitrogen, 10% hydrogen, 5% carbon dioxide) in sealed Gaspak jars containing Anaerogen packs. The bacterial counts were titrated on MRS agar plates and were expressed as colony-forming units (CFU) per ml as described previously [19]. The bacteria suspensions (in MRS with 15%~20% of glycerol) were stored at −80 °C. Before feeding, the bacteria were thawed and washed twice with 0.1% peptone water by centrifuging at 378× *g* (2000 rpm) for 10 min at 4 °C, and were diluted to specified CFU/mL.

### 2.3. Challenge Virus

The virulent HRV (VirHRV) Wa strain (G1P[8]) was passaged through Gn pigs, and the pooled intestinal contents from the 29th passage were used for challenge of Gn pigs at a dose of ~10^5^ fluorescence forming units (FFU) per pig. The 50% infectious dose (ID_50_) and 50% diarrhea dose (DD_50_) of the VirHRV in pigs were determined as approximately 1 FFU [20]. The virus titer was determined by using a cell culture immunofluorescence (CCIF) assay and was expressed as FFU/mL, as described previously [21].

### 2.4. Experimental Design: Gn Pig Inoculation, Challenge, and Sample Collection

Gn pigs were derived by hysterectomy from near-term sows (Landrace and Large White crossbred) and maintained in germ-free isolator units [22]. Pigs were fed commercial ultra-high temperature (UHT)-treated sterile milk. All pigs were confirmed as seronegative for rotavirus antibodies and germ-free prior to enrollment in the study. Pigs (both males and females) were randomly assigned to different treatment groups (Figure 1). A total of 31 pigs were included in the study. Gn pigs were orally inoculated with three doses of the TLV with (*n* = 7) or without (*n* = 9) LGG. Negative control groups received LGG alone (*n* = 8) or media alone (mock control) (*n* = 7). Pigs in LGG groups (TLV+LGG and LGG) were orally inoculated with 9 increasing doses (10^3^, 10^3^, 10^4^, 10^4^, 10^5^, 10^5^, 10^6^, 10^6^, and 10^6^ CFU) of LGG in 3 mL of 0.1% peptone water at post-partum day (PPD) 3, 4, 5, 6, 7, 8, 9, 10, and 11 once a day using a needle-less syringe. This LGG feeding regimen was selected based on our previous study that demonstrated effectiveness for enhancing the immunogenicity of the Wa AttHRV vaccine in Gn pigs [14]. Pigs in non-LGG fed groups (TLV and mock control) were given an equal volume of 0.1% peptone water. Gn pigs in TLV immunized groups (TLV, TLV+LGG), were orally inoculated with 1.8 × 10^6^ PFU of the TLV vaccine in 5 mL of diluent#5 (MEM with 1% non-essential amino acid, 1% antibiotic mixture [penicillin 10,000 I.U./mL, streptomycin 10,000 MCG/mL and amphotericin 25 MCG/mL]) at PPD 5 (post-TLV inoculation day (PID) 0), and boosted with the same dose at PPD 15 (PID 10) and PPD 26 (PID 21). Pigs in non-TLV inoculated groups (LGG and mock control) were given an equal volume of diluents#5.

At PID 28 (post-VirHRV challenge day (PCD) 0), subsets of pigs from all treatment groups were orally challenged with 1 × 10^5^ FFU of VirHRV in 5 mL of diluent#5. Pigs were given 8 mL of 100 mM sodium bicarbonate 20 min before virus inoculation or challenge to reduce gastric acidity. All inoculums were slowly instilled into the mouth at the back of the throat using a needleless syringe. Pigs were euthanized at PID 28 (PCD 0) or PID 35 (PCD 7). Serum, large intestinal content (LIC), and small intestinal content (SIC) samples were collected as depicted in Figure 1. Ileum, spleen, and blood samples were collected at euthanasia for isolation of mononuclear cells (MNCs), as previously described [14]. All animal experimental procedures were conducted in accordance with protocols reviewed and approved by Virginia Polytechnic Institute and State University’s Institutional Animal Care and Use Committee.

### 2.5. Clinical Signs and Rotavirus and Probiotic Bacteria Shedding

After the VirHRV challenge, pigs were examined daily from PCD 0 to PCD 7 for clinical signs, including % with diarrhea, duration of diarrhea, and fecal consistency (diarrhea scores), as previously described [15], and fecal and nasal swabs were collected daily for detection of virus shedding. The rotavirus infection in Gn pigs was confirmed by fecal virus shedding using enzyme-linked immunosorbent assay (ELISA) and CCIF assay, as previously described [14]. From PPD 2, fecal swabs were also collected weekly, diluted and plated on regular blood agar plates, and cultured aerobically at 37 °C for 24–72 h to check for the sterility, and later for the purity of colonizing bacteria of each Gn pig isolator.

### 2.6. Shedding of Vaccine Virus by RT-PCR Typing Primer for G2, G3, and G4

After TLV inoculation of Gn pigs, rectal swabs were collected daily from PID 2 to PID 27 for detection of replication of TLV in the gut. The total RNA was isolated from the swab samples with an RNeasy mini kit (Qiagen, Hilden, Germany), according to the user’s manual. RNA concentration was adjusted to 50 µg/µL as the template for reverse transcription. The shedding of the vaccine virus was detected by RT-PCR typing primers previously described [23]. Briefly, the sequence of the primers for the synthesis of VP7 cDNA was designed in the conserved regions of the 5′ and 3′ terminals of the VP7 gene. The sequences of type-specific primers were designed based on variable regions of VP7 gene (coding VP7 protein, with DS-1 for G2, SA11 for G3, and Hochi for G4), and unique in the LD9 (G2), LS4/9 (G3), and LH9 (G4) rotavirus reassortant strains. RNA from LD9 (G2), LS4/9 (G3), and LH9 (G4) virus strains, respectively, was used for primers’ screening and validation. The established RT-qPCR assays resulted in satisfying amplifications. The sequences of primers are listed in Table 1. The cDNA of VP7 gene was synthesized using M-MLV reverse transcriptase (Invitrogen, Waltham, MA, USA). The reaction system of reverse transcription was as follows: 0.2 µL of VP7 F primer (10 µM), 0.2 µL of VP7 R primer (10 µM), 5 µL of purified total RNA, 1 µL of 10 mM dNTP mix, 5.6 µL of RNase free water, heat mixture to 65 °C for 5 min, then quickly chill on ice. Collect the contents of the tube by brief centrifugation, then add the following components: 4 µL of 5× First-Strand Buffer, 2 µL of 100 mM DTT, 1 µL of ribonuclease inhibitor, and 1 µL of M-MLV, incubate at 37 °C for 50 min, heat the reaction at 70 °C for 15 min to inactivate the enzyme. The VP7 gene of G2, G3, and G4 reassortants was amplified using Platinum Taq DNA polymerase (Invitrogen) by type-specific primers, respectively. The reaction system is as follows: 5 µL of 10× high fidelity PCR buffer, 2 µL of 50 mM MgSO_4_, 1 µL of 10 mM dNTP, 2 µL of type-specific primer pairs (1 µL of forward primer and 1 µL of reverse primer), 0.2 µL of Platinum Taq DNA polymerase, 5 µL of cDNA and 34.8 µL of autoclaved distilled water. The incubation cycles were 94 °C 1 min, 72 °C 3 min; 94 °C 30 s, 56 °C 30 s, 72 °C 45 s (25 cycles); 72 °C 10 min. PCR products were subjected to electrophoresis in 2% agarose, and the target DNA band was imaged with a gel imager system (Bio-Rad).

### 2.7. Extraction of Mononuclear Cells (MNCs)

MNCs from the ileum, spleen, and peripheral blood were isolated as previously described [14]. Briefly, MNCs were extracted from the ileum by using EDTA twice and collagenase twice and enriched by discontinuous Percoll gradient, from the spleen by mechanical separation and enriched by discontinuous Percoll gradient, and from blood by using Ficoll-Paque^TM^ plus.

### 2.8. Preparation of Sf9 Cell Plates Expressing Rotavirus D VP7 (G1), DS1 VP7 (G2), PV P7 (G3), and ST3 VP7 (G4)

Separate cultures of Sf9 cells in Grace’s medium (10% FBS) were infected with recombinant baculovirus expressing rotavirus D VP7 (G1), DS1 VP7 (G2), P VP7 (G3), and ST3 VP7 (G4), respectively. The infected Sf9 cells were transferred to 96-well plates using 5 × 10^5^ cells/well, and plates were incubated at 27 °C for 40 h until cells expressed the corresponding individual rotavirus proteins. The cell culture medium was carefully removed so as not to disrupt the Sf9 cell monolayer, and the plates were air dried. The infected Sf9 cell monolayers were fixed by adding 4% formaldehyde at room temperature for 30 min, as described previously [24]. The fixed cells were permeabilized with 1% Triton X-100 in TNC buffer (10 mM Tris-HCl, 140 mM NaCl, 10 mM CaCl_2_ (pH 7.5)) at room temperature for 10 min. The plates were stored at −20 °C up to 7 days before use. The plates were used to examine rotavirus VP7-specific ASCs by ELISPOT assay and serum antibody by the immunocytochemical staining assay, as previously described [25].

### 2.9. Immunocytochemical Staining Assay for Detection of Rotavirus G1, G2, G3, and G4 VP7 G-Type-Specific IgA and IgG Antibody Responses in Serum

The titer of serum IgA and IgG antibodies to rotavirus G1, G2, G3, and G4 VP7 proteins in TLV, TLV+LGG immunized pigs were measured using immunocytochemical staining assay as previously described [24]. Briefly, Sf9 cell plates infected with recombinant baculovirus expressing rotavirus D VP7 (G1), DS1 VP7 (G2), P VP7 (G3), and ST3 VP7 (G4), respectively, were washed with deionized water before use. Sera were assayed in 4-fold serial dilution. Antibodies that bound to Sf-9 cells on the plates were detected using an anti-porcine IgG-Fc antibody HRP conjugate (Bethyl, Inc, Montgomery, TX, USA) and an anti-porcine IgA antibody HRP conjugate (Bethyl, Inc). Sf-9 cells stained with the primary and the secondary antibodies were visualized with AEC substrate (3-amino-9ethylcarbazole; Sigma, St. Louis, MO, USA). The antibody titer was defined as the reciprocal of the highest dilution at which any positive cell staining could be detected under the microscope at 100× magnification. To ensure that variations in the amount of the individual rotavirus proteins expressed in insect cells do not affect the accuracy of the test, serial 4-fold dilutions of monoclonal (mAb) or polyclonal antibodies directed to each expressed viral protein were included on each plate as internal positive controls. The test plates were used only when the titer variation of the mAb or hyperimmune antiserum was within a 4-fold dilution. In addition, data were accepted for analysis only when the titers of the positive controls were consistent on all plates for each viral protein.

### 2.10. ELISPOT Assay for Rotavirus G1, G2, G3, G4 VP7 G-Type-Specific ASC

The extracted MNCs were subjected to an enzyme-linked immunospot (ELISPOT) assay immediately after isolation for the enumeration of ASC. ELISPOT assays to enumerate rotavirus VP7-specific ASC were conducted by using previously published methods [25]. Briefly, recombinant baculovirus expressing rotavirus D VP7 (G1), DS1 VP7 (G2), P VP7 (G3), and ST3 VP7 (G4) infected Sf9 cell plates, respectively, were washed with deionized water prior to use. Cell suspensions of MNC from each tissue were added to duplicate wells (5 × 10^5^, 5 × 10^4^, and 5 × 10^3^ cells/well) and centrifuged at 500× *g* for 30 min to ensure the cell sedimentation onto the monolayer and the production of optimal spots. Plates were incubated for 12 h at 37 °C in 5% CO_2_ and then washed and incubated with HRP conjugated pig IgA and pig IgG-Fc antibody diluted 1: 20,000 in 1% fat-free dry milk in PBS. After incubating the plate at 37 °C for 2 h, the plates were washed to remove the cells, and spots were developed with TMB TrueBlue (KPL Inc, Geithersburg, MD, USA). The numbers of rotavirus VP7-specific ASC were determined by counting blue spots in the wells and were reported as the number of rotavirus VP7 G-type-specific ASC per 5 × 10^5^ MNC.

### 2.11. Intracellular Cytokine Staining and Flow Cytometry Analysis of IFN-γ-Producing CD4+ and CD8+ T Cells

Flow cytometry was used to determine frequencies of rotavirus VP7-specific and non-specific IFN-γ-producing CD4+ and CD8+ T cells in the ileum, spleen, and blood of Gn pigs, as we previously described [26]. Briefly, the purified MNCs from all tissues were resuspended in the complete medium consisting of RPMI-1640 (Gibco, BRL, Geithersburg, MD, USA) supplemented with 8% fetal bovine serum, 20 mM HEPES (N-2-hydroxyethyl-piperazine-Nk-2-ethane sulphonic acid), 2 mM L-glutamine, 1 mM sodium pyruvate, 0.1 mM non-essential amino acids, 100 μg/mL of gentamicin, 10 μg/mL of ampicillin, and 50 mM 2-mercaptoethanol (E-RPMI). The cells were restimulated in vitro with 12 μg/mL of semi-purified Wa AttHRV antigen (G1), reassortant rotavirus LD (G2) antigen, reassortant rotavirus LS (G3) antigen, reassortant rotavirus LH (G4) antigen, positive control PHA (10 μg/mL), or mock stimulated (E-RPMI only) for 17 hrs at 37 °C. Brefeldin A (10 μg/mL; Sigma, St. Louis, MO, USA) was added for the last 5 hrs to block the secretion of cytokines produced by the T cells. For flow cytometry, all antibodies and reagents were titrated and used at optimal concentrations. The appropriate isotype-matched irrelevant antibody controls were included for MNCs from each tissue in each staining as negative controls to set the quadrant markers for the bivariate dot plots. The second set of controls was stained with all the antibodies to surface markers and the cytokine antibody was replaced with the isotype control antibody. At least 100,000 cells were acquired on a FACSAria flow cytometer (BD Biosciences, San Jose, CA, USA). Data were analyzed using FlowJo 7.2.2 software (Tree Star, Ashland, OR, USA). The frequencies of IFN-γ+CD4+ and IFN-γ+CD8+ T cells were expressed as percentages among total CD3+ T cells. All mean frequencies were reported after subtraction of the background frequencies.

### 2.12. Statistical Analysis

Means of ASC counts and mean T-cell responses were compared among treatment groups using the non-parametric Kruskal–Wallis rank sum test followed by Dunn’s multiple comparisons. In the case when only two groups were compared, the Mann–Whitney test was used. Proportions of affected animals (infected and with diarrhea) among treatment groups were compared using Fisher’s exact test. The area under the curve (AUC) of diarrhea score and virus shedding were calculated in RStudio using the AUC command of the DescTools package and the spline approach. Mean day to onset, duration of virus shedding and diarrhea, and mean AUC of virus shedding and diarrhea among the treatment groups were compared using one-way ANOVA or Kruskal–Wallis rank sum test when the normality and homoscedasticity assumptions were not met. The IgA and IgG antibody titers in serum from PID 0 to PID 35/PCD 7 and the virus shedding titer and diarrhea score from PCD 1–7 were analyzed by a two-way ANOVA of repeated measures through time. Correlation between the AUC of virus shedding and the IgG, IgA, and VN antibody titers in serum were studied by linear regression analysis. Statistical significance was assessed at *p* < 0.05. Statistical analyses were performed using GraphPad Prism Version 9 from GraphPad Software, LLC (San Diego, CA, USA) or RStudioVersion 1.4.1717 from RStudio, PBC.

## 3. Results

### 3.1. Vaccine Replication in Gn Pigs: Shedding of G3 and G4 Reassortant Strains was Detected after Oral Inoculations with TLV+LGG

**Figure 2:**
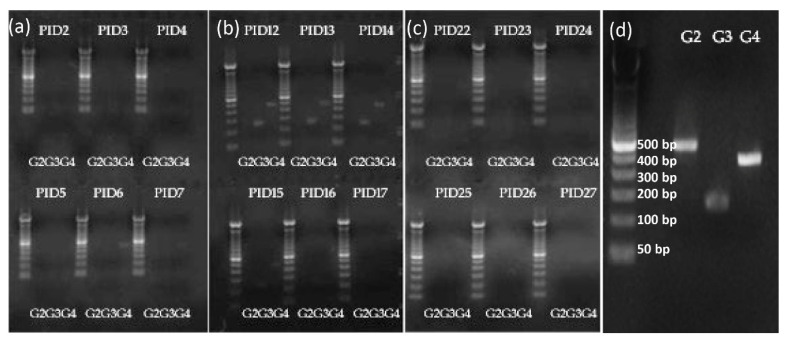
Representative images of fecal virus shedding were detected by VP7-specific RT-PCR. The shedding of reassortant virus strains in TLV from the Gn pigs in the TLV+LGG group was monitored after the oral administration of each vaccine dose. (**a**) Fecal swabs collected after dose 1 inoculation; (**b**) fecal swabs collected after dose 2 inoculation; (**c**) fecal swabs collected after dose 3 inoculation; (**d**) RT-PCR amplification of G2, G3, and G4 DNA fragments from mixed positive control by using rotavirus VP7-specific G2, G3, and G4 typing primers detailed in Table 1.

To evaluate the replication capacity of the different attenuated reassortant human x lamb virus strains of the TLV in the presence of LGG in Gn pigs, virus shedding in the LGG+TLV group was monitored by G-typing RT-PCR for 6 days after the oral administration of each of the three vaccine doses (Figure 2). The G4 strain was detected in 100% (3/3) of pigs on PID 6 and 33% (1/3) of pigs on PID 5 and PID 7 after the first dose. Both G3 and G4 strains were detected in 100% of pigs on PID 12 and 67% on PID 13–15. No virus shedding was detected from PID 16–17 or after the 3rd dose vaccination from PID 22–27. G2 was not detected at any of the time points tested.

### 3.2. LGG Enhanced TLV Vaccine’s Protection against Heterotypic Virulent Wa HRV Challenge

**Figure 3:**
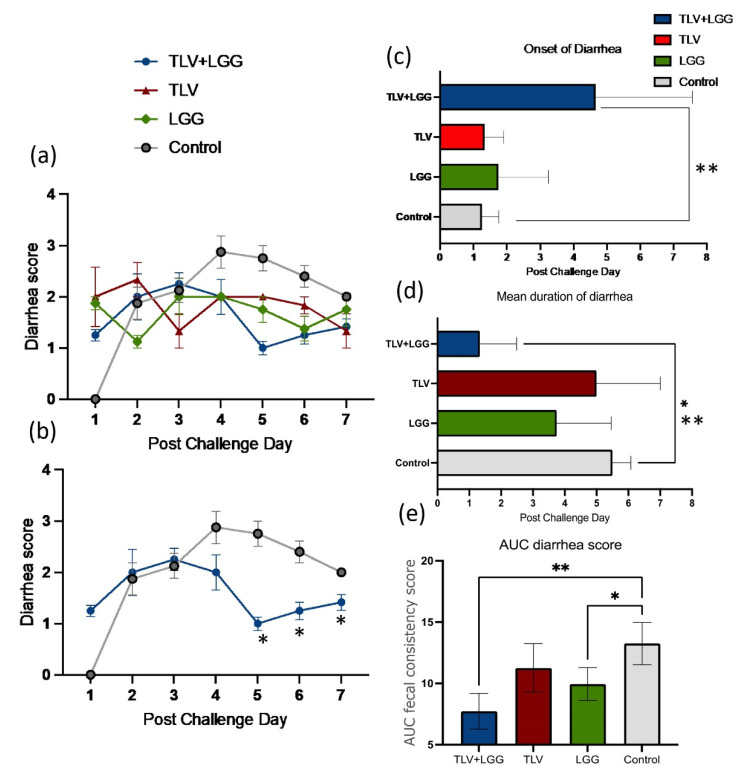
Protection against diarrhea upon virulent Wa HRV challenge. Pigs were challenged orally with virulent Wa HRV at PID 28 and monitored for 7 days post-challenge (PCDs 1–7) for duration and severity of diarrhea. * Indicates significant difference when comparing between two groups; ** indicates significant difference between the TLV+LGG group and all the other groups. (**a**) Mean diarrhea score from PCD 0 to 7 in all treatment groups. (**b**) Mean diarrhea score of TLV+LGG versus the control. (**c**) Mean days to onset of diarrhea. (**d**) Mean duration of diarrhea. (**e**) Mean area under the curve of diarrhea score.

Significant protection from diarrhea and virus shedding against heterotypic challenge with virulent Wa HRV were conferred by the LGG-adjuvanted TLV vaccine in Gn pigs. The TLV+LGG group had a lower percentage of diarrhea than the TLV, LGG, and mock control groups (67% vs. 100%). TLV+LGG vaccinated pigs exhibited a significant delay in the mean onset of diarrhea, significantly shorter mean duration of diarrhea, and significantly lower mean AUC of diarrhea scores and mean cumulative diarrhea scores compared to the pigs in the TLV, LGG, and mock control groups (Table 2 and Figure 3c–e). LGG-fed pigs also had significantly lower AUC of diarrhea scores than the control pigs (Figure 3e). When comparing the daily diarrhea scores, pigs in the TLV+LGG, TLV, and LGG groups had overall lower diarrhea scores from PCDs 4–7 than the control group (Figure 3a). The TLV+LGG vaccinated pigs had significantly lower daily diarrhea scores on PCDs 5–7 compared to the control pigs (Figure 3b).

**Figure 4:**
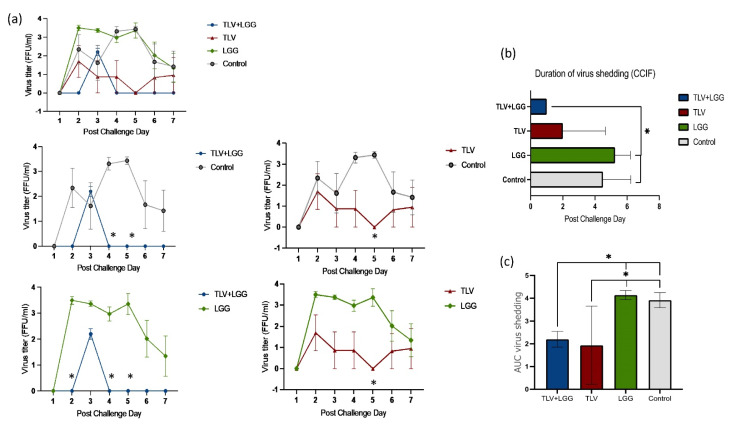
Protection against virus shedding upon virulent Wa HRV challenge. Pigs were challenged orally with virulent Wa HRV at PID 28 and monitored 7 days postchallenge (PCDs 1−7) for duration and magnitude of virus shedding by CCIF. * Indicates significant difference when compared with the LGG or control group. (**a**) Mean virus shedding from PCDs 1 to 7 compared among all groups or compared pairwise between different groups. (**b**) Mean duration of virus shedding measured by cell culture immunofluorescence (CCIF). (**c**) Mean area under the curve (AUC) of virus shedding in each treatment group.

Regarding protection against infection, pigs in all groups shed virus after challenge, with similar mean days to onset (Table 2). However, both the TLV+LGG and TLV groups had overall lower titers of virus shedding from PCDs 4–7 than the LGG and control groups (Figure 4a). Pair-wise comparisons revealed significantly reduced virus shedding titers in the TLV+LGG and TLV groups compared to the LGG and control groups on PCDs 4–5 and PCD 5, respectively (Figure 4a). The TLV+LGG group had a significantly shorter mean duration of virus shedding and significantly lower AUC of virus shedding compared to the LGG and control groups (Table 2 and Figure 4b,c). The TLV group also had a significantly lower AUC of virus shedding than the LGG and mock control groups (Figure 4c). These data demonstrated that heterotypic protection against virus shedding was conferred by the TLV vaccine, and the LGG adjuvant further enhanced the protective effect.

### 3.3. The TLV Induced Intestinal VP7-Specific IgA and IgG ASC Responses to Homotypic and Heterotypic VP7 G-Types; The Presence of LGG Promoted Stronger Intestinal Vaccine Antigen-Specific ASC Responses

The Gn pigs in the two non-immunized groups (LGG and mock control) did not develop VP7-specific IgA and IgG ASC responses before challenge (PID 28/PCD 0) or 7 days post-challenge (PCD 7), which is as expected (Table S1).

**Figure 5:**
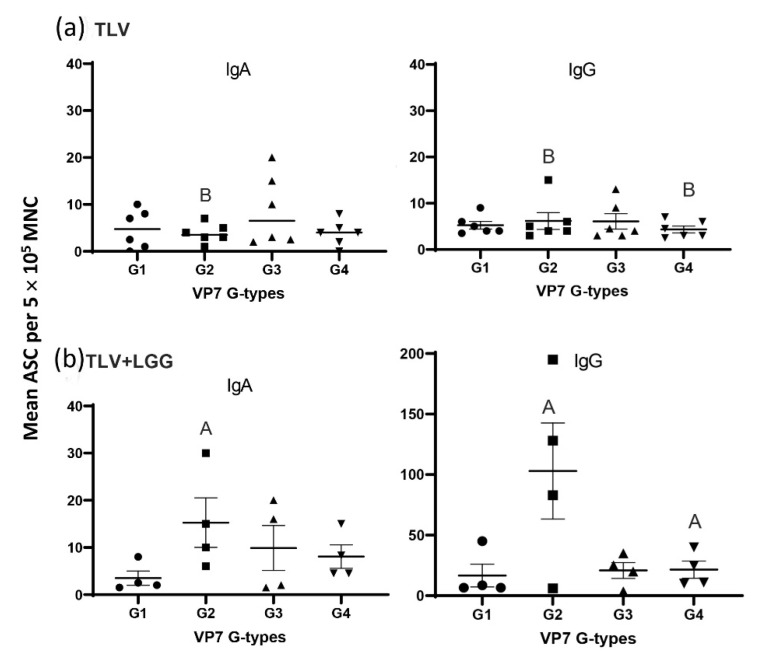
IgA and IgG VP7 G-type-specific ASC responses in the ileum at challenge (PID 28/PCD 0) in (**a**) pigs orally immunized with TLV vaccine, and (**b**) pigs immunized with TLV vaccine in the presence of LGG. The ASC responses to different VP7 G-types were statistically similar within each vaccine group (Kruskal–Wallis non-parametric rank sum test, *p* < 0.05). Mean in the same column with different capital letters indicates significant differences between the TLV and TLV+LGG groups for the same G type. Lines and error bars represent the mean and SEM.

VP7 G-type specific IgA and IgG ASC responses in the small intestine (ileum) at PID 28/PCD 0 (Figure 5a). The concomitant oral administration of the TLV and LGG induced significantly higher counts of IgA ASC for G2, and IgG ASC for G2 and G4 VP7 G-types than TLV alone (Kruskal–Wallis, non-parametric rank sum test, *p* < 0.05) (Figure 5a,b).

**Figure 6:**
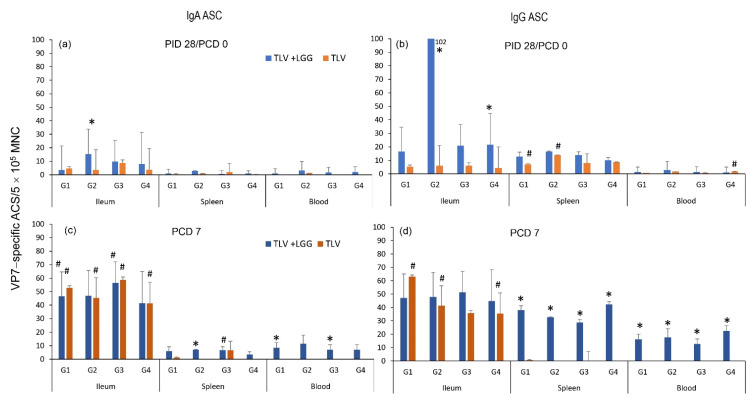
IgA and IgG VP7–specific ASC responses in the intestinal and systemic lymphoid tissues prechallenge (PID 28/PCD 0) (a and b) and postchallenge (PCD 7) (c and d). ^#^ Indicates a significant difference between PID 28/PCD 0 and PCD 7 for each vaccine group in each tissue for each VP7 G–type * Indicates a significant difference between TLV+LGG and TLV groups for the same VP7 type in the same tissue and timepoint (Kruskal–Wallis non–parametric rank sum test, *p* < 0.05). Pigs in the LGG and mock control groups did not develop VP7–specific ASC responses (Table S1).

When analyzing the IgA and IgG VP7-specific ASC responses pre- and post-challenge with the G1P[8] virulent Wa HRV in intestinal (ileum) and systemic lymphoid tissues (spleen and blood) (Figure 6), the IgA responses were mainly detected in the ileum, corresponding to the replication site of the vaccine, whereas IgG responses were detected in both intestinal and systemic lymphoid tissues. There were no or very low IgA and IgG ASC counts in the spleen and blood for both vaccinated groups at PID 28/PCD 0. The LGG adjuvant significantly increased the number of G2 VP7-specific IgA and IgG ASC in the ileum and G4 VP7-specific IgG ASC in the ileum when compared with the pig receiving the vaccine alone pre-challenge. Post-challenge, both vaccinated groups developed strong anamnestic IgA and IgG responses in the ileum, with higher or significantly higher numbers of ASC at PCD 7 compared to PCD 0 for all the four VP7 G-types. The TLV + LGG pigs developed significantly higher IgG ASC responses to all four VP7 types in the spleen and blood than the TLV pigs at PCD 7. Overall, the ASC responses induced by the TLV vaccine were enhanced or significantly enhanced by the concurrent administration of LGG, in all tissues tested and at both timepoints, not only for all the VP7 G-types present in the vaccine, but also for the heterotypic G1P[8] HRV used for the challenge (Figure 6).

### 3.4. LGG Adjuvant Enhanced VP7 G-Type Specific IgA and IgG Antibody Responses in Serum Pre- and Postchallenge, including the Cross-Reactive Response to the Heterotypic G1 HRV Challenge Strain

**Figure 7:**
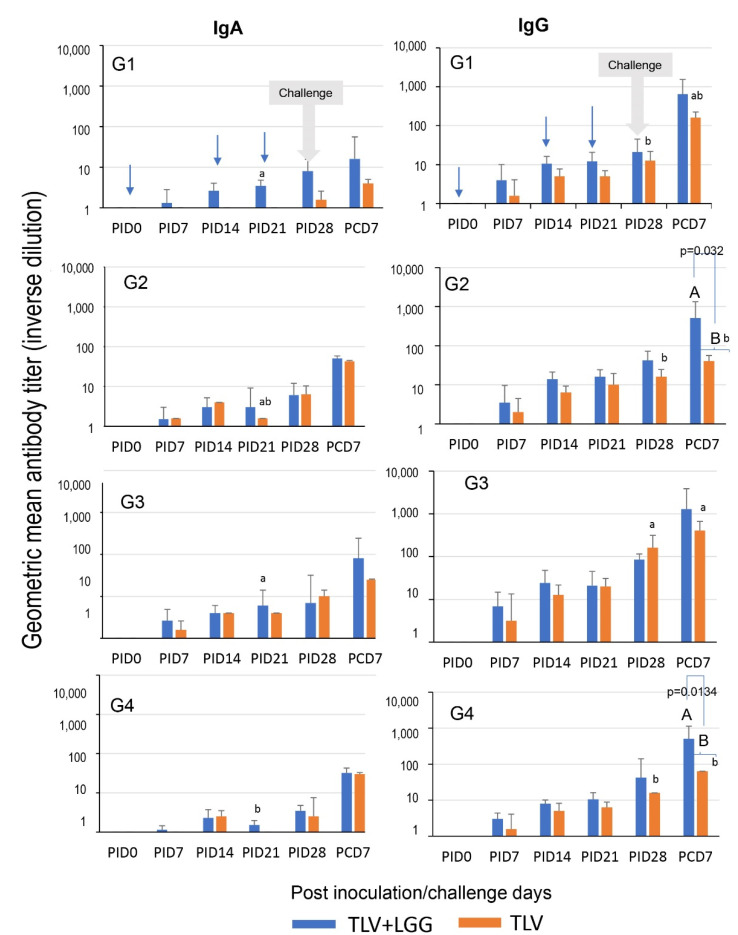
VP7 G-type specific IgA (**left** panel) and IgG (**right** panel) antibody responses detected by immunocytochemical staining assay in serum of Gn pigs vaccinated with TLV with or without LGG. Bars with different uppercase letters (A, B) indicate a statistical difference between treatment groups (repeated measure ANOVA-GLM, *p* < 0.05). Bars with different lowercase letters (a, b) indicate significant differences among VP7 G-type specific antibody responses within each vaccine in the selected timepoint (one-way ANOVA, *p* < 0.05). Blue arrows indicate the immunization times (3 ×) and grey arrows the challenge time with virulent Wa HRV G1P[8] strain.

The kinetics of G1, G2, G3, and G4 VP7–specific IgA and IgG antibody responses in the serum of pigs immunized with the TLV with or without the administration of LGG are depicted in Figure 7. In concordance with the ASC results, the TLV alone (orange bars) induced low levels of IgA responses to the three G–type VP7s in the vaccine (G2, G3, and G4) from PID 14 onward, and low cross-reactive IgA to G1 at PID 28. In contrast, high IgG antibody responses to all four VP7 G–types were detected from PID 14 onward.

When comparing the serum IgA and IgG responses to different VP7 G-types in the TLV alone group, the antibody responses to G3 were higher than the other G-types throughout, with significantly higher titers at challenge (PID 28) and post-challenge (PCD 7) (Figure 7; lowercase letters, ANOVA-GLM, *p* < 0.05; *n* = 3–6).

The coadministration of LGG and TLV (blue bars) increased serum VP7-specific IgA and IgG antibody titers to all VP7 G-types pre- and postchallenge, resulting in significantly higher titers of IgG to G2 and G4 than those of the TLV alone at PCD 7 (Figure 7; uppercase letters, repeated measure ANOVA-GLM, *p* < 0.05; *n* = 3–6). This is consistent with the LGG’s stimulating effect on G2- and G4-specific ASC responses in the intestinal lymphoid tissue at PID 28.

Within the TLV+LGG group, the IgA and IgG titers were statistically similar among all G-types at all timepoints, except for IgA at PID 21 where G1 and G3 antibody titers were significantly higher than those for G4 (Figure 7; lower case letters, repeated measures ANOVA-GLM, *p* < 0.05; *n* = 3–6).

### 3.5. The TLV Primed for Strong Anamnestic Serum VN Antibody Responses to Heterotypic G1P[8] HRV Post-Challenge with or without LGG

**Figure 8:**
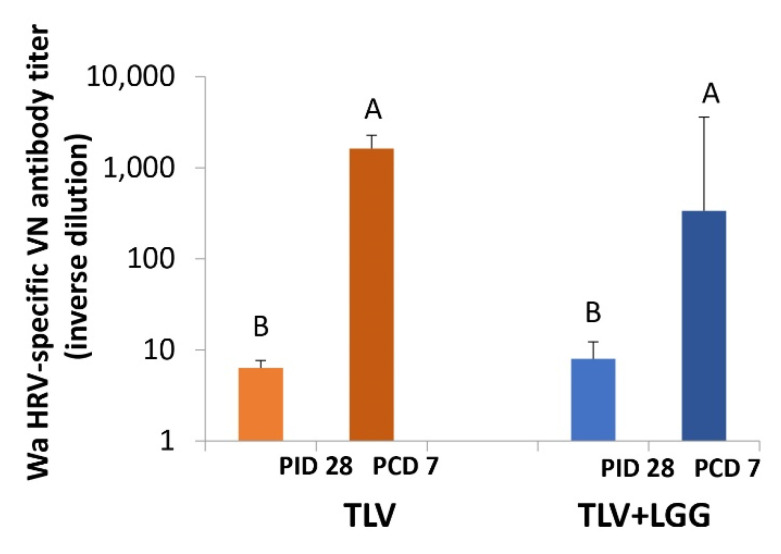
Virus neutralizing antibody responses to Wa HRV pre- and postchallenge in TLV and TLV+LGG groups. Bars with different uppercase letters (A, B) differs significantly. Antibody titers between the two groups on each timepoint were not significantly different. VN antibody titers of both groups increased significantly postchallenge (repeated measure ANOVA-GLM, *p* < 0.05).

The TLV induced low serum VN antibody titers to the heterotypic G1P[8] HRV Wa strain at PID 28. The concomitant administration of LGG did not improve the VN titers. After challenge, both vaccine groups showed a significant increase from PID 28 to PCD 7 in the Wa HRV-specific VN titers, indicating the priming for cross-reactive anamnestic responses. Final titers were statistically similar between both groups (Figure 8, repeated measure ANOVA-GLM, *p* < 0.05).

### 3.6. IFN-γ-Producing CD4+ and CD8+ T Cell Responses in Intestinal and Systemic Lymphoid Tissues

#### 3.6.1. Both LGG and TLV Alone Induced IFN-γ-Producing CD4+ and CD8+ T-Cell Responses in Intestinal and Systemic Lymphoid Tissues

**Figure 9:**
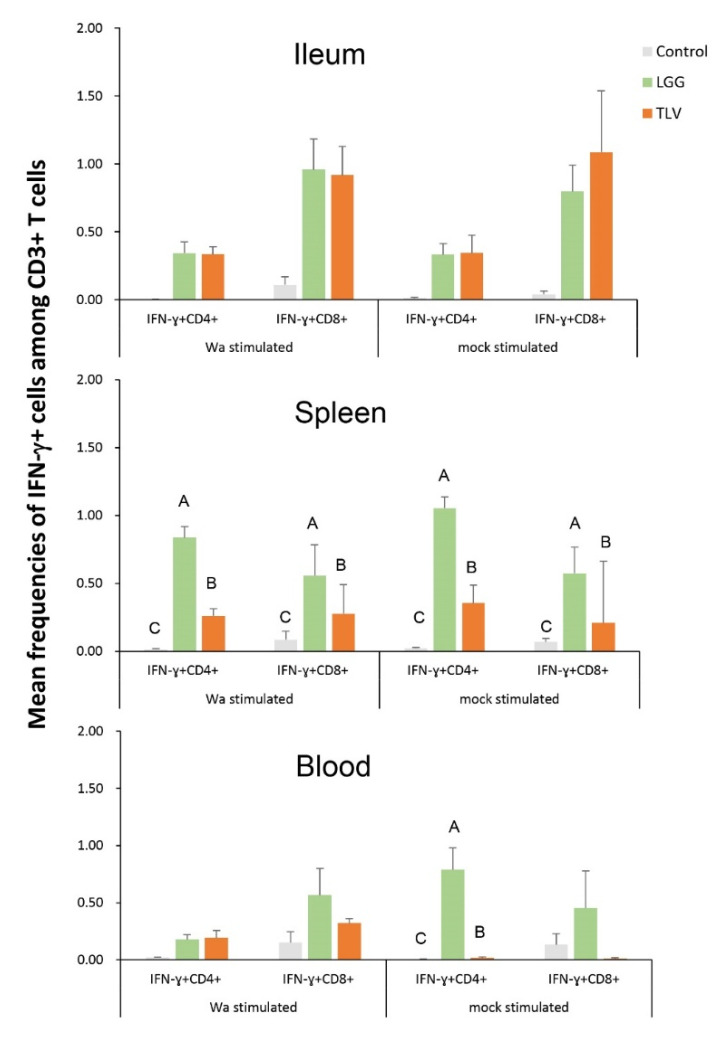
Frequencies of IFN-γ+CD4+ and IFN-γ+CD8+ T cells in the intestinal and systemic lymphoid tissues detected by flow cytometry pre-challenge (PD 28/PCD 0) in the LGG-fed, TLV-vaccinated, and mock control pigs. The MNC were stimulated in vitro for 17 h with semi-purified whole Wa HRV antigen or mock stimulated. Capital letters on top of bars (A, B, C) indicate significant difference, whereas shared letters indicate no difference among the groups for the same cell type (Kruskal–Wallis test, *p* < 0.05).

First, we assessed whether the LGG or TLV alone can induce effector T cell responses to the heterotypic challenge virus. We used semi-purified Wa HRV antigen or mock antigen to stimulate the MNCs in vitro. LGG induced significantly higher frequencies of IFN-γ+CD4+ and IFN-γ+CD8+ T cells in the spleen and IFN-γ+CD4+ in blood than the TLV and controls, regardless of antigen stimulation. Compared to the pigs in the mock control group, TLV-vaccinated pigs had higher or significantly higher frequencies of IFN-γ+CD4+ and IFN-γ+CD8+ T cells in ileal, splenic and blood MNCs stimulated with either Wa HRV, or mock antigen, demonstrating the induction of viral-specific and no-specific IFN-γ producing T-cell responses in the intestinal and systemic lymphoid tissues (Figure 9).

#### 3.6.2. The TLV Induced Heterotypic G1- and Homotypic G2-, G3- and G4-Specific IFN-γ-Producing CD4+ and CD8+ T-Cell Responses in Intestinal and Systemic Lymphoid Tissues Pre- and Postchallenge

**Figure 10:**
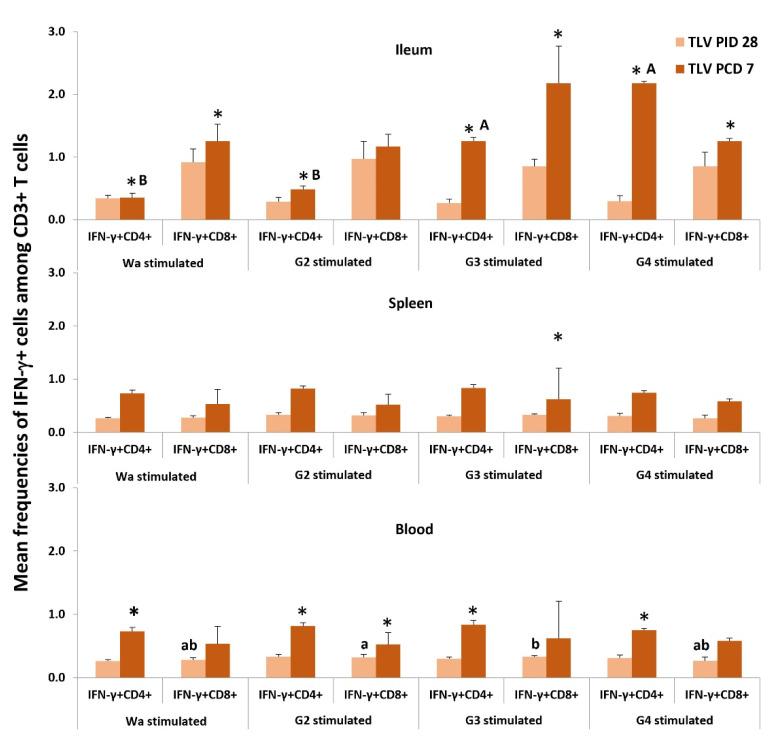
Frequencies of IFN-γ+CD4+ and IFN-γ+CD8+ T cells in the intestinal and systemic lymphoid tissues pre- and postchallenge in the TLV vaccinated Gn pigs detected by flow cytometry. The MNC were stimulated in vitro for 17 h with semi-purified whole Wa HRV, G2, G3, or G4 VP7 antigens. * Indicates a significant difference compared between PID 28 and PCD 7 for the same cell type. Different letters on the bars indicate significant differences between different antigen stimulation for IFN-γ+CD4 T cells (capital letters A, B) and IFN-γ+CD8 T cells (lowercase letters a, b); while shared letters indicate no significant difference at the same time point (Kruskal–Wallis test, *p* < 0.05).

Second, we examined the induction by TLV alone of heterotypic G1 (Wa)- and homotypic G2-, G3- and G4 -specific IFN-γ-producing CD4+ and CD8+ T-cell responses (Figure 10). IFN-γ+CD4+ and IFN-γ+CD8+ T cells were detected with similar frequencies among MNCs stimulated with the four types of viral antigens in the spleen and blood at PID 28. Frequencies of IFN-γ+CD4+ and CD8+ T cells increased or significantly increased (indicated by *) from PID 28 to PCD 7 in all three tissues for all four antigen stimulations. There were no major differences among the different antigen stimulations. In the ileum, frequencies of IFN-γ+CD4+ T cells in G3- and G4-stimulated MNCs were significantly higher than G1 and G2 at PCD 7, suggesting a stronger anamnestic T-cell response to G3 and G4 post-challenge.

#### 3.6.3. The LGG Modulates IFN-γ-Producing CD4+ and CD8+ T-Cell Responses Differentially in Intestinal and Systemic Lymphoid Tissues

**Figure 11:**
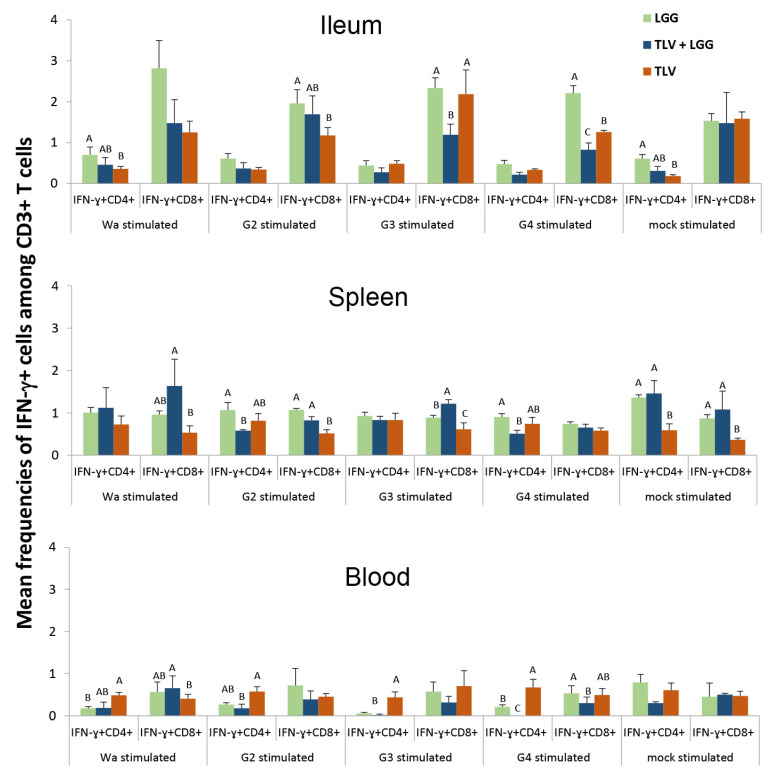
Frequencies of IFN-γ+CD4+ and IFN-γ+CD8+ T cells in the intestinal and systemic lymphoid tissues of Gn pigs in the TLV+LGG, TLV, and LGG groups at PCD 7 detected by flow cytometry. The MNC were stimulated in vitro for 17 h with semi-purified whole Wa HRV, G2, G3 and G4 VP7 antigens, or mock stimulated. Bars with different uppercase letters (A, B) indicate significant differences among groups for the same cell type, while shared letters indicate no difference (Kruskal–Wallis test, *p* < 0.05).

To evaluate the interactions among LGG, TLV, and HRV challenge on IFN-γ producing T cell responses, frequencies of IFN-γ+CD4+ and CD8+ T cells in LGG alone, TLV alone, and TLV+LGG pig groups at PCD 7 were compared (Figure 11).

In the ileum, LGG alone induced overall the highest frequencies of IFN-γ+CD4+ and IFN-γ+CD8+ T cells. The frequencies of IFN-γ+CD4+ T cells were similar between the TLV+LGG and TLV groups and across the different antigen stimulations. The IFN-γ+CD8+ T cell responses to G3 and G4 antigen in the ileum were significantly lower in the TLV LGG group compared to the TLV or LGG group. In the spleen, frequencies of IFN-γ+CD4+ T cells in G2 and G4 antigen-stimulated MNCs of the TLV+LGG group were significantly lower than those of the LGG group, whereas they were significantly higher in mock-stimulated MNCs of the TLV+LGG and LGG groups than the TLV group. The frequencies of IFN-γ+CD8+ T cells in Wa, G2, G3, and mock antigen-stimulated splenic MNCs of the TLV+LGG group were significantly higher than the TLV group. In blood, TLV alone induced significantly higher IFN-γ+CD4+ T-cell responses than the TLV+LGG and LGG for Wa, G2, G3, and G4 antigen-stimulated MNCs. IFN-γ+CD8+ T-cell responses in the TLV+LGG group trended lower compared to the TLV group for G2, G3, and G4 antigen-stimulated MNCs.

## 4. Discussion

The Lanzhou Lamb RV (LLR) vaccine is a live oral rotavirus vaccine consisting of the attenuated LLR strain (serotype G10P[12]) that has been licensed in China for domestic application since 2000. The vaccine is administered in one dose in children between 6 and 12 months of age, followed by yearly boosters. Even with excellent overall performance, due to the lack of reduction of RVA detection rate in ambulatory patients and the emergence of different strains including the G9P[8] combination that has become the prevalent strain in the recent year in China, an alternative improved RV vaccine was developed by Lanzhou Institute of Biological Products Co., Ltd., in China. The trivalent Lanzhou TLV consists of three rotavirus reassortants, LD, LS, and LH strains, that were generated using HRV field isolate D36 (G2), S25 (G3), and the standard Hochi (G4) strain as VP7/VP4 gene donor strains, and the LLR strain as the backbone strain. The present study is a comprehensive preclinical evaluation of the mucosal and systemic immune responses induced by the TLV vaccine in a gnotobiotic pig model of rotavirus infection and disease and a proof of principle of how the concurrent administration of the probiotic adjuvant LGG can significantly improve the immunogenicity and protection of this oral vaccine against heterologous challenge with a G1P[8] HRV strain.

In this study, we first demonstrated the replication of the different RV strains included in the TLV vaccine in the presence of LGG in the gut of the Gn pigs. After oral immunization, the shedding of each vaccine strain was evaluated by G-type specific RT-PCR. Vaccine shedding was detected in 100% of the Gn pigs on PID 6 after the first dose; the timing indicates that these viruses were not the passing-through vaccines. In addition, even administered in similar titer, the replication of the different G-types was different in Gn pigs. The shedding of G3 and G4 strains was detected by RT-PCR, while the G2 strain was not. Some speculations about the reasons for this differential replication pattern might be that G3XLLR reassortant (LS) carries both VP4 (P [2]) and VP7 (G3) gene segments from the HRV donor strain; this may explain why this strain replicated well in the Gn pigs and induced overall the highest viral-specific immune responses. G2XLLR (LD) carries one gene segment, VP7, from the HRV donor strain, D36, and the rest of the 10 segments from LLR which has the genotype constellation (G10-P [15]-I10-R2-C2-M2-A11-N2-T6-E2-H3), typical for bovine-like rotavirus strains. Therefore, it was not unexpected that G2XLLR replication in Gn pigs was not detected and the immune responses to G2 VP7 were the lowest among the three VP7 types because G2XLLR does not carry any gene segments from porcine-like rotavirus (the VP7 gene donor strain D36 is also a bovine-like rotavirus). The observed vaccine shedding in Gn pigs agrees with the vaccine shedding reported in a field trial in human infants, where 14% (16/114) of the vaccinated children shed vaccine RV for at least 1 day from PID day 2 to 13, and peaked on PID 5 to10 [27]. The absence of fecal vaccine shedding following the third dose of the vaccine indicates the intestinal mucosal immunity generated by the first two doses in Gn pigs, and concurs with what has been reported in human infants after the first dose of Rotarix vaccination [28].

Second, our study demonstrated that heterotypic protection was conferred by the TLV vaccine, and the LGG adjuvant further enhanced the protective effect. Although the TLV or TLV+LGG did not significantly prevent the % of pigs developing diarrhea or virus shedding upon challenge with the Wa HRV, TLV+LGG strongly reduced the severity of diarrhea among affected animals, as evidenced by the significantly reduced AUC of diarrhea and the improvement of all parameters (onset days, mean duration, mean cumulative score, and daily score) compared to the control pigs. LGG alone also significantly reduced the AUC of diarrhea. LGG’s effect on rotavirus diarrhea has been documented in our previous studies in Gn pigs [29] and in many human clinical studies [30]. LGG and TLV demonstrated an additive effect in reducing diarrhea against the Wa HRV challenge, similar to the previously observed additive effect of LGG and the attenuated Wa HRV vaccine (AttHRV+LGG) against the homotypic virulent Wa HRV challenge [14]. TLV with or without LGG significantly reduced the AUC of virus shedding compared to LGG alone and the control, indicating vaccine-induced cross-G type virus-specific immunity inhibited virus replication. Furthermore, the TLV+LGG group had the shortest mean duration of virus shedding, 2.3 days shorter than the TLV alone. All pigs in the TLV+LGG group shed low titers of the Wa HRV for only one day post-challenge on PCD 3. The quick clearance of virus infection indicates the enhanced anti-viral immunity by the LGG adjuvant.

Corresponding to the cross G-type protective effects, the TLV vaccine induced homotypic and heterotypic intestinal and systemic effector B and T cell and antibody immune responses; the LGG adjuvant further enhanced these immune responses pre- and post-challenge. Assessment of VP7 G-type specific ASC responses showed that TLV induced similar levels of homotypic (G2, G3, and G4) and heterotypic (G1) IgA and IgG ASC responses in the ileum, and LGG significantly enhanced the numbers of IgA and IgG ASC to G2 VP7 in the ileum before the challenge. Since the G2 vaccine virus replicated the poorest among the three reassortants (shedding was not detectable by RT-PCR), LGG’s adjuvant effect is the most significant for G2. Equally notable is that LGG enhanced systemic IgG ASC responses. The TLV+LGG pigs had significantly higher IgG ASC responses to all four VP7 types in the spleen and blood than the TLV pigs at PCD 7. Increasing evidence has shown that orally administered probiotic bacteria can stimulate the immune system, both at the gut and at distant sites. Through the production of cytokines, probiotics trigger the stimulation of adaptive immune responses and establish a network of signals among the different immune cells that can circulate from the intestinal to the systemic lymphoid tissues [31]. Although LGG is a pro-Th1 probiotic strain [12] and induced strong IFN-γ-producing T-cell responses at the dosage we used in the Gn pigs, LGG did not negatively affect the induction of mucosal IgA ASC responses, agreeing with the findings in a study of human microbiota transplanted pigs [32]. Not only LGG has been tested in numerous randomized clinical trials for reducing the severity of diarrhea [33,34,35,36,37], but clinical studies also demonstrated that LGG promoted antibody production [38,39,40]. LGG enhanced rotavirus-specific IgA ASC responses in human adults [38] and rotavirus-specific IgM ASC responses and IgA seroconversion in 2- to 5-month-old infants [41].

The TLV vaccine alone induced strong and broadly reactive IgG antibody titers in serum in concordance with the ASC responses registered in the ileum and spleen. Low serum IgA titers were detected after three TLV doses for all G-types (GMT: G1 = 1.6, G2 = 6.3, G3 = 10, G4 = 2.5). However, it is important to highlight that the results observed in Gn pigs were in the same range as the IgA GMT obtained when the TLV vaccine (named LLR3) was tested in a clinical trial in human infants (GMT: G2 = 2.27, G3 = 1.99, G4 = 2.38), where the vaccine efficacy against RV gastroenteritis of any severity and caused by any serotype was 56.6% [5]. The concurrent administration of the LGG adjuvant significantly improved the antibody responses induced by the TLV, as evidenced by the improved kinetics of IgA and IgG antibody responses to each VP7 G-type in serum of the TLV+LGG pigs; for IgA, it is characterized by an earlier detection at PID 7, and for IgG, it is the higher titers. This result highlights the importance of considering the addition of probiotics to the TLV oral vaccine formulation to promote fast and enhanced IgA and IgG ASC and antibody responses that are crucial for vaccine efficacy. In this regard, a randomized, double-blind, placebo-control trial of the pentavalent RotaTeq vaccine conducted in China showed higher efficacy than the TLV [7,42]. The RotaTeq vaccine induced higher anti-RV IgA (82.42 units/mL, 89.4%) responses compared to the placebo group (0.33 units/mL, 10.1%), which were associated with a 69.3% vaccine efficacy against RV gastroenteritis of any severity caused by any serotype.

Regarding the development of VN antibodies to the heterotypic challenge virulent Wa HRV strain, it is important to note that both TLV and TLV+LGG induced statistically similar, low VN titers pre-challenge that were significantly increased post-challenge. Interestingly, the improvement in the efficacy of TLV observed by the co-administration of LGG against the G1 HRV challenge was not associated with an increase in the G1-specific VN antibody titers. VN antibody titers were also not associated with protective immunity in live oral vaccine evaluations in animals [43] and human clinical trials [44,45]. However, when analyzing the relationship between the serum antibody responses and the virus shedding across all the treatment groups, we observed a significant inverse correlation between the IgA, IgG, and VN antibody titers in serum at challenge (PCD 0) of each individual Gn pig and the AUC of virus shedding, indicating that these parameters measurable from blood samples are good markers of protection against infection (Figure S1).

Virus-specific intestinal IFN-γ-producing T cells are known to correlate with protection against HRV diarrhea in Gn pigs [26]. The T-cell responses likely played a role in the observed protective immunity induced by the TLV. We observed cross G-type reactive (Wa-specific), as well as non-specific IFN-γ-producing T-cell responses in pigs that received TLV alone at PID 28, demonstrating the effective priming of effector T-cell immune responses by the TLV. We also observed strong anamnestic intestinal and circulating IFN-γ+CD4+ and CD8+ T-cell responses across all four G types in the TLV vaccinated pigs from PID 28 to PCD 7. Strikingly, LGG alone induced similar frequencies of IFN-γ+CD4+ and CD8+ T cells to those of the TLV alone in Wa-stimulated and mock-stimulated MNCs in the ileum, and higher or significantly higher frequencies in the spleen and blood at PID 28. The T-cell responses in the TLV+LGG group at challenge were not measured, but an additive or synergistic effect could be expected based on the significantly early virus clearance in the TLV+LGG pigs than in the TLV or LGG alone pigs. The IFN-γ-producing T cells activated by LGG alone likely contributed to the protective effect in reducing diarrhea and promoted systemic antibody production. However, unlike TLV, LGG alone did not significantly reduce virus shedding.

The pattern of T cell responses observed in immunized pigs post-challenge is very complex because multiple contradicting factors contribute to the composition. The pigs who have complete intestinal immunity and are able to totally prevent virus replication would have no or very low virus antigen re-exposure upon challenge, hence no or very low anamnestic T-cell responses. However, even in the same treatment group, the pigs who have stronger but incomplete immunity may have lower or higher IFN-γ-producing T-cell responses than the pigs who have weaker immunity and are subjected to stronger virus replication, hence more antigen re-exposure, depending on the level of immunity and the time course of virus replication in the individual pigs post-challenge. This complex T-cell response is further compounded by the influence of LGG’s stimulation on IFN-γ-producing T-cell responses in the TLV+LGG group. The comparisons of T-cell responses among the LGG alone, TLV alone, and TLV+LGG groups at PCD 7 in an effort to evaluate the influence of LGG on TLV-induced T-cell responses reflected such complexity. The only clear and consistent finding is that TLV+LGG pigs had overall significantly higher frequencies of IFN-γ+CD8+ T cells in the spleen, corresponding to the significantly higher IgG ASC responses in the spleen than the TLV pigs at PCD 7, indicating the enhancement of systemic cellular and humoral immunogenicity of the TLV by LGG.

In summary, the three doses of oral TLV were highly immunogenic and conferred partial protection against heterotypic HRV infection, and LGG enhanced the intestinal and systemic immune responses and improved the effectiveness of protection against heterotypic HRV diarrhea and virus shedding in orally vaccinated Gn pigs. Thus, LGG served as an effective mucosal adjuvant for the oral rotavirus vaccine. LGG is one of the best-studied probiotics in clinical trials [46]. Due to the low cost, ease of distribution and administration, and favorable safety profiles, LGG as an adjuvant has the potential to play a critical role in improving rotavirus vaccine efficacy in places where the vaccine efficacy is less than optimal, and in reducing the dose and dosing requirement of rotavirus vaccines to make the vaccines more abundantly available, affordable, and cost-effective.

To translate the findings of LGG as an adjuvant in our studies to human clinical use, further experiments are needed. The Gn pig model allows us to clearly see the effect of the probiotic adjuvant in the absence of other microbiota components that may act as a confounding variable in the immunomodulation of the immune response to vaccination, but it does not represent the real-life situation of vaccinating children that possess variable microbiota. In this regard, a meta-analysis conducted by Zimmermann and Curtis [11] summarized the immunomodulatory effects of probiotics observed in randomized placebo-controlled studies conducted in humans. They surveyed a total of 26 studies, involving 3812 participants, investigating the use of 40 different probiotic strains on the efficacy of 17 different vaccines. A beneficial effect of probiotics was reported in half of the studies. The positive effect was especially clear in oral vaccines. The authors discussed that the favorable effect might be due to the modification of the microbiota, promoted by the probiotics. These results indicate that the effect described in Gn pigs can be extrapolated to human infants. However, concurrent vaccine efficacy studies in children, Gn pigs, and Gn pigs transplanted with human microbiota could be an important experiment to conduct in the future to define the mechanism behind probiotics’ immunomodulatory effects [11]. An important point to highlight is that probiotics offer a relatively cheap intervention to improve vaccine efficacy and duration of protection that will be especially helpful in developing countries.

## 5. Conclusions

In conclusion, through this study, we demonstrated the immune-stimulating effects of probiotic LGG as a vaccine adjuvant and generated detailed knowledge regarding the cross-reactive and type-specific antibody and effector B- and T-cell immune responses induced by the TLV. The knowledge will facilitate the understanding of the mechanisms of homotypic versus heterotypic immunity induced by rotavirus vaccines. The most significant finding from this study is that, with the probiotic LGG as an adjuvant, there was clear heterotypic protection observed. The role of gut microbiota in vaccine efficacy is increasingly being recognized, and using probiotic adjuvants to modulate the gut microbiota to enhance vaccine efficacy has been poised to be the next frontier of vaccine development.

## Figures and Tables

**Table 1:** Sequence typing primer for G2, G3, and G4 RVs.

Primers	Name	Sequence (5′→3′)
Primers for VP7 gene reverse transcription	VP7-F	tct ggc taa cgg tta gct cc
	VP7-R	aat act tgc cac cat ttt ttc c
Specific primers for G2	G2-F	caa tga tat taa cac att ttc tgt
	G2-R	aat act tgc cac cat ttt ttc c
Specific primers G3	G3-F	ctt ttg aag aag ttg cga cag
	G3-R	aat act tgc cac cat ttt ttc c
Specific primers for G4	G4-F	cgc ttc tgg tga gga gtt g
	G4-R	aat act tgc cac cat ttt ttc c

**Table 2:** Clinical signs and rotavirus fecal shedding in Gn pigs after virulent Wa human rotavirus (HRV) challenge.

Treatments	*n*	Clinical Signs (Diarrhea)					Fecal Virus Shedding			
		% with Diarrhea *^,a^	Mean Days to Onset **^, b^	Mean Duration Days **	Mean AUC of Diarrhea Score ^c^	Mean Cumulative Score **	% Virus Shedding *	Mean Days to Onset **^,b^	Mean Duration Days **	Mean AUC of Virus Shedding ^d^
TLV+LGG	3	100 ^A^	4.7 (1.7) ^fA^	1.3 (0.7) ^B^	7.7 (0.8) ^C^	9.3 (0.8) ^B^	100 ^A^	2.0 (0.3) ^A^	1.0 (0) ^B^	2.2 (0.2) ^B^
TLV	3	67 ^A^	1.3 (0.3) ^B^	5.0(1.2) ^A^	11.3 (1.1) ^AB^	12.8 (1.6) ^A^	100 ^A^	1.7 (0.3) ^A^	3.3 (0.7) ^AB^	1.9 (0.9) ^B^
LGG	4	100 ^A^	1.8 (0.8) ^B^	3.8 (0.9) ^A^	9.9 (0.7) ^B^	11.9 (0.8) ^A^	100 ^A^	2.0 (0) ^A^	5.3 (0.5) ^A^	4.2 (0.1) ^A^
Mock control	4	100 ^A^	1.3 (0.3) ^B^	5.5 (0.3) ^A^	13.2 (0.9) ^A^	16.9 (1.3) ^A^	100 ^A^	2.0 (0.3) ^A^	4.5 (0.9) ^A^	3.9 (0.2) ^A^

^a^ Pigs with daily fecal scores ≥ 2 were considered diarrheic. Fecal consistency was scored as follows: 0, normal; 1, pasty; 2, semiliquid; and 3, liquid; ^b^ If the pigs did not develop diarrhea or virus shedding until euthanasia day (postchallenge day [PCD] 7), the days to onset diarrhea or virus shedding was recorded as 8 for statistical analysis; ^c^ Mean of the area under the curve (AUC) of diarrhea score from PCD 1 to PCD 7; ^d^ Mean AUC of virus shedding from PCD 1 to 7, measured by cell culture immunofluorescent (CCIF) assay or antigen enzyme-linked immunosorbent assay (ELISA), were expressed as the log10 transformed titer in FFU (fluorescent focus forming units)/mL; ^f^ Standard error of the mean; * Proportions in the same column with different superscript letters (^A, B^) differ significantly (Fisher’s exact test, *p* ≤ 0.05); ** Mean in the same column with different superscript letters (^A, B, C^) differ significantly (Kruskal–Wallis or one-way ANOVA, Dunn’s multiple comparison test comparing all groups vs. control, *p* ≤ 0.05).

## Data Availability

Data supporting reported results were all generated during the study and presented in this manuscript.

## Supplementary Materials

The following supporting information can be downloaded at: https://www.mdpi.com/article/10.3390/vaccines10091529/s1, Figure S1: Linear regression analysis between the AUC of virus shedding after challenge with Wa G1P[8] HRV and the antibody titers in serum at PID 28/PCD 0; Table S1: Mean number of VP7-specific IgA and IgG in intestinal and systemic tissues are compared at challenge (PCD 0) and postchallenge (PCD 7) in each treatment group.
