# Supplementary material for: Probiotic as Adjuvant Significantly Improves Protection of the Lanzhou Trivalent Rotavirus Vaccine against Heterologous Challenge in a Gnotobiotic Pig Model of Human Rotavirus Infection and Disease
Source: Vaccines (Basel). 2022 Sep 14;10(9):1529. doi: 10.3390/vaccines10091529 (PMC9506166; doi:10.3390/vaccines10091529)
Supplement: Supplementary file 1 [file vaccines-10-01529-s001.zip › vaccines-1900043-supplementary.pdf]

Suplementary material

Table S1. Mean number of VP7-specific IgA and IgG ASC/5x10^5 MNC in intestinal and systemic tissues are compared at challenge (PCD 0) and postchallenge (PCD 7) in each treatment group.

Treatment group	Timepoint	n	Mean IgA ASC VP7-specific/5x10^5 MNC G1	G2	G3	G4	Mean IgG ASC VP7-specific/5x10^5 MNC G1	G2	G3	G4
**Ileum**										
TLV 3x+LGG	PID35/PCD7	6	46.7 A	47	56.6 A	41.5 A	47.2	48	51.4	44.8
	PID28/PCD0	4	3.5 B	15	9.8 B	8.1 B	16.7	103	20.9	21.5
	p value		*0.019*	*0.8095*	*0.0381*	*0.4429*	*0.5619*	*0.3143*	*0.5048*	*0.6381*
TLV 3x	PID35/PCD7	3	53 A	45.3 A	58.7 A	41.3 A	63.0 A	41.3 A	35.7	35.3 A
	PID28/PCD0	6	4.8 B	3.6 B	8.8 B	3.8 B	5.3 B	6.1 B	6.1	4.3 B
	p value		*0.0238*	*0.0238*	*0.0238*	*0.019*	*0.019*	*0.019*	*0.068*	*0.049*
LGG	PID35/PCD7	4	0	0	0	0	0	0	0	0
	PID28/PCD0	4	0	0	0	0	0	0	0	0
MOCK	PID35/PCD7	4	0	0	0	0	0	0	0	0
	PID28/PCD0	4	0	0	0	0	0	0	0	0
**Spleen**			**G1**	**G2**	**G3**	**G4**	**G1**	**G2**	**G3**	**G4**
TLV 3x+LGG	PID35/PCD7	6	6.0	7.1	6.8 A	3.5	38.1	32.7	28.8	42.3
	PID28/PCD0	4	1.0	2.8	0.6 B	0.9	12.9	16.5	13.9	10.0
	p value		*0.587*	*0.591*	*0.0415*	*0.398*	*0.199*	*0.668*	*0.108*	*0.39*
TLV 3x	PID35/PCD7	6	1.3	0	6.7	0.0	0.7 B	0 B	0.3 B	0 B
	PID28/PCD0	3	0.5	1	2	0.3	7.2 A	13.9 A	8.0 A	8.8 A
	p value		*0.261*	*0.317*	*0.796*	*0.317*	*0.046*	*0.037*	*0.046*	*0.036*
LGG	PID35/PCD7	3	0	0	0	0	0	0	0	0
	PID28/PCD0	4	0	0	0	0	0	0	0	0
MOCK	PID35/PCD7	4	0	0	0	0	0	0	0	0
	PID28/PCD0	4	0	0	0	0	0	0	0	0
**Blood**			**G1**	**G2**	**G3**	**G4**	**G1**	**G2**	**G3**	**G4**
TLV 3x+LGG	PID35/PCD7	6	8.6	12	7	7.0	16.3	17.6	12.75	22.4
	PID28/PCD0	4	0.9	3	1.9	2.0	1.4	2.8	1.5	1.1
	p value		*0.061*	*0.666*	*0.139*	*0.379*	*0.285*	*0.334*	*0.08*	*0.104*
TLV 3x	PID35/PCD7	3	0	0	0	0.0	0	0	0	0.0
	PID28/PCD0	3	0	1.3	0.2	0	0.8	1.7	0.8	1.8
	p value		*na*	*na*	*0.317*	*na*	*0.121*	*0.121*	*0.121*	*0.036*
LGG	PID35/PCD7	3	0	0	0	0	0	0	0	0
	PID28/PCD0	4	0	0	0	0	0	0	0	0
MOCK	PID35/PCD7	4	0	0	0	0	0	0	0	0
	PID28/PCD0	4	0	0	0	0	0	0	0	0

The ASC counts at PCD 0 vs. PCD 7 were compared by Kruskal Wallis non-parametric rank sum test (Mann-Whitney for two groups), $p<0.05$, (package Agricolae, R Studio). Means in the same column with different letter, differ significantly.
The control group were not included in the analysis since no ASC were detected at both timepoints.

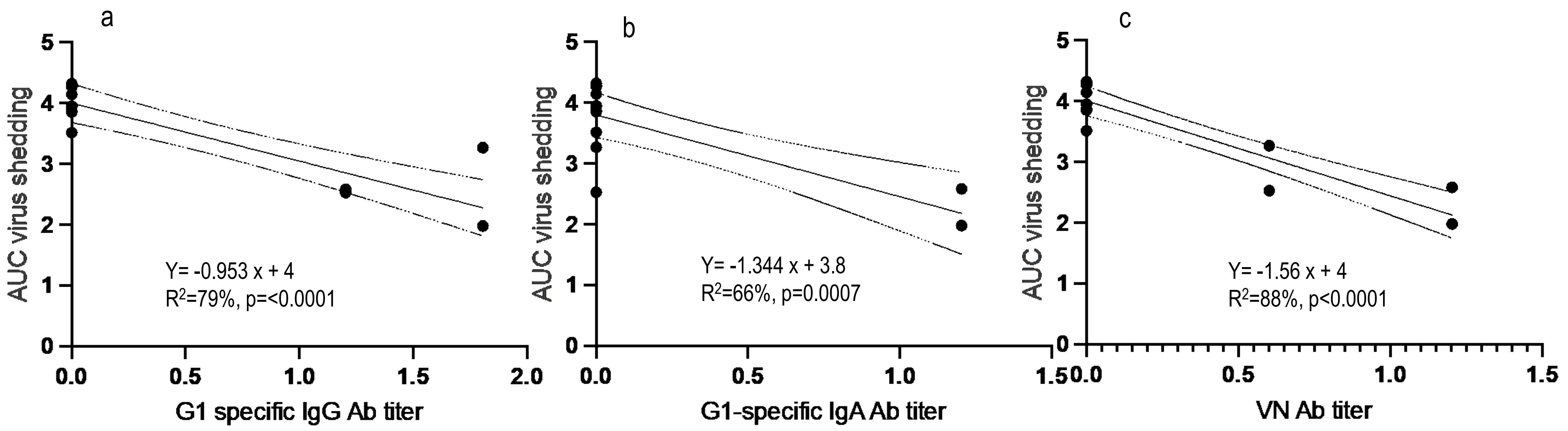

Figure S1. Linear regression analysis between the AUC of virus shedding after challenge with Wa G1P[8] HRV and the antibody titers in serum at PID28/PCD0. a) G1-specific IgG Ab titers, b) G1-specific IgA Ab titers, and c) virus neutralizing Ab titers. Dots represent values from each animal; lines depict the means predicted by the model with the upper and lower 95% CI bands.
